# Primary Stability of a New TendoClip Fixation for Tuberosity Refixation in Total Shoulder Arthroplasty

**DOI:** 10.1002/jor.70105

**Published:** 2025-12-13

**Authors:** Maximilian Uhler, Thilo Patzer, Jörg Fleischer, Daniel Knopp, Kevin Knappe, Matthias Bülhoff, Jan Philippe Kretzer

**Affiliations:** ^1^ Research Center of Biomechanics and Implant Technology, Clinic for Orthopedics and Trauma Surgery University Hospital Heidelberg Heidelberg Germany; ^2^ Department for Shoulder‐Elbow Surgery and Sports Orthopedics Schoen Clinic Duesseldorf Duesseldorf Germany; ^3^ Department for Traumatology and Orthopedics Hospital Marienhaus St. Wendel‐Ottweiler St. Wendel Germany; ^4^ Heinrich‐Heine‐University of Duesseldorf Duesseldorf Germany; ^5^ Department of Orthopaedics University Hospital Heidelberg Heidelberg Germany

**Keywords:** in vitro investigation, proximal humeral fracture, reverse shoulder arthroplasty, tuberosity reattachment

## Abstract

Sufficient refixation and healing is crucial for the function of a reverse shoulder arthroplasty within the treatment of proximal humeral fractures. Conventional refixation with suture cerclage is time‐consuming and leads to healing in only 60–70% of cases. This study used a biomechanical test to investigate two different refixation procedures for treating 4‐fragment fractures of the proximal humerus. Eight paired humerus specimens were randomly assigned to two groups and treated with reverse shoulder prostheses. One group received conventional suture cerclage fixation for 4‐fragment fractures, while the other used a novel TendoClip system with screw fixation. Both groups were subjected to increasing cyclic tensile forces until failure. Fragment movement relative to the prosthesis stem was measured to assess primary stability. Tensile forces generated in the first four load levels resulted in significantly different relative motions between the TendoClip and cerclage groups. 3 out of 8 specimens in the TendoClip group could be loaded over all load levels. This was not possible for any cerclage group specimens. Recorded failure mechanisms showed that fixed bone fragments of the cerclage procedure were affected significantly more often by loosening and visible relative movements compared to the TendoClip procedure. However, limitations of the TendoClip restoration were also observed. In 2 of 8 cases, a bone fragment was torn out of the fixation, and two other implants showed a loss of fixation in relation to the humerus. This study reveals that the TendoClip procedure could be an alternative to conventional cerclage fixation procedures in multifragmentary proximal humerus fractures.

## 1 Introduction

Proximal humerus fracture (PHF) is a prevalent traumatic injury of the upper extremity [1, 2]. Different treatment methods are used depending on the severity of the fracture and its classification, e.g. according to Neer et al. [3] but these methods are still not strictly defined. Unstable and dislocated extra‐articular fractures are usually surgically reduced and stabilized. There is no evidence for an ideal procedure to treat 4‐fragment fractures. However, an endoprosthetic treatment is mostly considered in the case of osteoporosis or small and dislocated fragments. This applies especially to elderly patients with displaced PHF, with reverse shoulder arthroplasty (RSA) taking precedence over hemiarthroplasty [4]. Compared to hemiarthroplasty, RSA is characterized by a better functional and clinical outcome [5, 6] but can lead to a reduction in active rotation [7]. This disadvantage can be overcome by reattaching the tuberosities to the prosthesis. Healing of the tuberosities, including muscle attachments in their anatomical position, can significantly improve rotation [8]. Even if the healing of the tuberosities is not ideal, there is an improved functional outcome compared to HA without reattachment [9, 10, 11].

Besides the functional outcome benefits, proper tuberosity healing, including muscle attachments, also influences joint stability in RSA. The functional rotator cuff can increase compression forces, which can reduce possible shear forces [12, 13]. This can minimize the risk of complications, such as instability of the prosthesis and loosening of the humeral component [14].

Nevertheless, refixation and with that the consecutive healing of the tuberosities with the attached tendons, is still not performed in a standardized manner using various suture fixation techniques. Healing rates averaging 71% were reported in a review study [15], with new prosthesis designs averaging 77% [16]. This means that over 20% of tuberosity fixations still fail. Insufficient attachment of the tubercula, a poor bone healing rate, or too early and high patient activity may lead to mal‐unions and non‐unions of both tubercles [17, 18].

The fixation techniques and materials used for reattachment of the tuberosity in RSA resemble those used in HA. Strong, non‐resorbable sutures or flexible titanium alloy cables are commonly applied to perform the cerclage‐like technique to treat the fracture [19, 20]. Biomechanical investigations of these techniques reveal further potential for improvement [21, 22, 23]. At this point, the increased risk of obstruction of the bony consolidation during refixation of tuberosities is particularly important [24].

**Figure 1:**
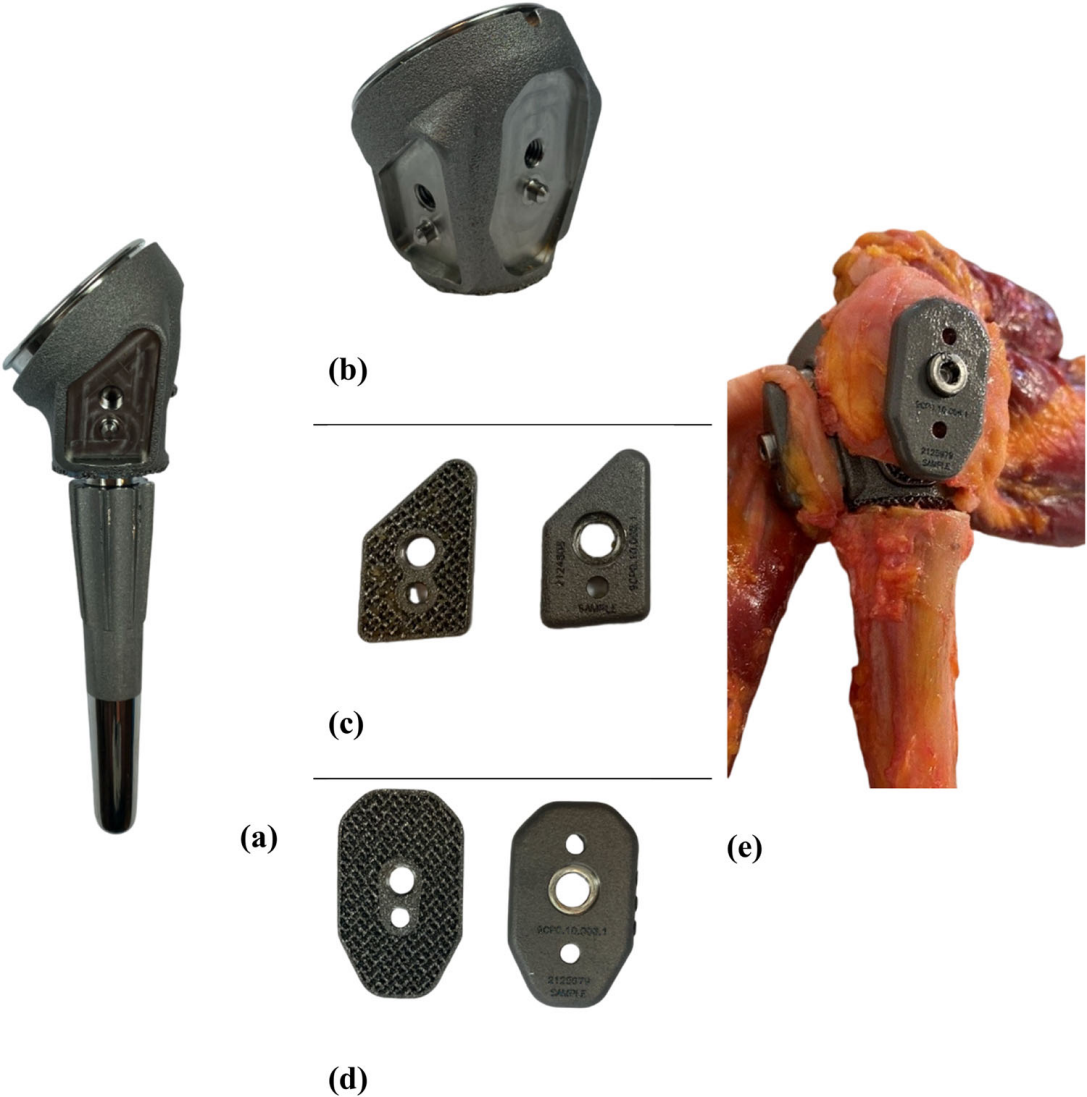
(a) Reverse shoulder endoprostheses with TendoClip body, (b) Novel prosthesis body for TendoClip fixation, (c, d) TendoClips for the lesser and greater tuberosities, (e) Surgical treatment with TendoClips.

With the aim of improving fixation, a new design for tuberosity fixation that is standardized and easy to perform was developed for anatomical and reverse shoulder endoprosthesis. It uses a so‐called TendoClip made of titanium (Figure 1). These clips clamp the tuberosity between them like a sandwich. The plates with the inserted tuberosity are then fixed to the metaphyseal prosthesis body with a central screw. TendoClip plates are made of textured, rough titanium on the bone side for better bone adhesion.

This study made an experimental biomechanical comparison of a standardized 3D suture cerclage with the TendoClip on a 4‐part PHF model treated with RSA. The rotational and translational implant migration in all three axes was determined by measuring the relative motion between the tuberosity fragments, implant, and bone. Our hypothesis is that due to the planar and screw‐retained connection of the tuberosity to the prosthesis, the primary stability of the new TendoClip under cyclic dynamic loading is significantly higher than the conventional 3D suture cerclage fixation technique. This also applies to the maximum failure load.

## 2 Materials and Methods

The local ethics committee approved this in vitro study using cadaveric specimens (Ethikkommission der Medizinischen Fakultät Heidelberg, reference S‐330/2022). The study was performed in accordance with the Declaration of Helsinki. Fresh‐frozen tissue samples were purchased from Science Care (Phoenix, AZ, USA), which is accredited by the American Association of Tissue Banks. A complete donor report, including each specimen′s medical history, was available before sample acquisition. Exclusion criteria were a history of shoulder injury or surgery, bone tumors or other malignant diseases in the shoulder part, serological concerns, and high‐performance sport. Biomechanical properties were preserved before the experimental periods by frozen storage [25].

Eight paired fresh‐frozen human shoulders (4 females; 4 males; 16 specimens) were purchased for the experimental investigation. Mean donor data were: age 55.4 ± 6.5 years, weight 68.3 ± 27.6 kg, height of 169.4 ± 6.7 cm, and body mass index 23.9 ± 8.1 kg/m². The right and left sides of the 8 shoulder pairs were randomly allocated to the cerclage and TendoClip groups through a computer‐generated list (Randlist 1.2; Datinf GmbH, Tübingen, Germany). Both groups were treated with a reverse shoulder endoprosthesis from Lima (SMR Reverse, Limacorporate S.p.A., Udine, Italy), whereby the prosthesis shaft is identical and the prosthesis body differs in the two different types of treatment.

Bone mineral density (BMD) was assessed for all 16 specimens to improve comparability. As there are no standardized examination procedures for the upper arm, the analysis was performed according to Doetsch et al. [26]. BMD was measured using standard dual‐energy absorptiometry (DXA) with standard hip parameters (Hologic QDR‐2000, Marlborough, Massachusetts, USA). The region of interest (ROI) was delimited by the proximal area of the humerus and the joint space between the glenoid and humeral head. ROI size was defined as the same for all samples. For all 16 shoulder joints, native radiographs in anterior‐posterior and lateral projections were obtained to exclude bone pathology and determine prosthesis size using TraumaCAD software (Voyant Health Ltd., Brainlab AG, Munich, Germany). Since the right and left humeri sizes did not differ considerably, the same prosthesis size was planned, and an optimal fit was provided for both sides of each shoulder pair.

### 2.1 Specimen Preparation

Specimens were prepared by removing the skin and soft tissue. Humeri were dissected while preserving the rotator cuff tendons inserting to the greater tuberosity (M. subscapularis), lesser tuberosity (M. infraspinatus) and tendon of the M. supraspinatus.

A standardized 4‐part proximal humerus fracture was created using an oscillating saw. The fracture model described by Charles Neer was used for standardization and reproducibility of the fracture model [3]. First, fracture lines were cut between the lesser and greater tuberosities and the humerus shaft. The fracture line was placed lateral to the intertubercular sulcus. The lesser and greater tuberosity were separated from the shaft. Care was taken not to injure the muscle tendons and to preserve the entire tendon to bone insertions of the rotator cuff tendons. For optimal alignment of the arthroplasty, the humerus was initially not shortened and the distal elbow joint was preserved.

### 2.2 Implantation and Tuberosity Fixation

All 16 treatments were performed by the same surgeon with original surgical instruments. First, the humeral stem was implanted with a press‐fit according to the manufacturer′s instructions. The prosthesis system has a modular design and is available for two structurally different prosthesis bodies. The humeral shaft does not differ. After the humeral shaft component was implanted, the corresponding prosthesis body was attached according to the randomization.

**Figure 2:**
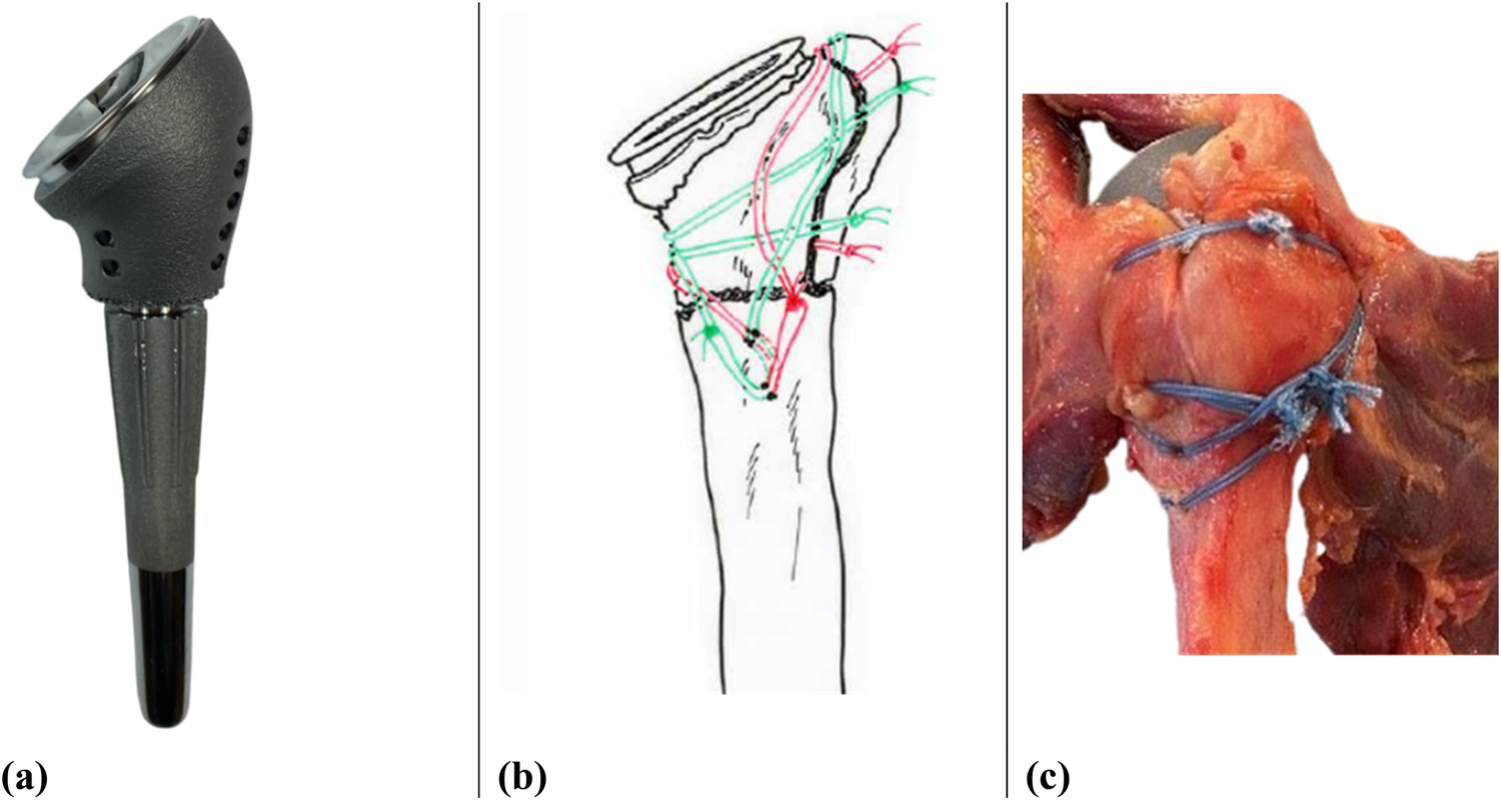
(a) Reverse shoulder endoprostheses with cerclage body, (b) standardized cerclage treatment, (c) Surgical treatment with cerclage.

The standard prosthesis body for the cerclage restoration has passages at defined increments to adequately fix the bony fragments (Figure 2a). In accordance with the surgical instructions, the greater tuberosity was first fixed in the correct anatomical position with two sutures. They were passed through the infraspinatus and supraspinatus tendon to bone insertion and the passages of the prosthesis body. The lesser tuberosity was then also fixed to the prosthesis body as well as the greater tuberosity, which was fixed with two sutures through the subscapularis and infraspinatus tendon. This ensures horizontal fixation by four double stranded cerclage sutures, two around the greater tuberosity and the reverse humeral body and two around the greater and lesser tuberosity and the reverse humeral body. For the final fixation and vertical support, two doubled sutures, which were passed through drill holes in the humerus, were applied. Once was placed clockwise through the lower subscapularis, upper subscapularis and supraspinatus tendon and once counterclockwise through the infraspinatus, supraspinatus and upper subscapularis tendon around the attached tuberosities. Non‐resorbable polyethylene sutures (Fiberwire 5, Arthrex, Naples, USA) were doubled up, tensioned manually, and tied by applying the Nice knot [27] for the applied fixation. The cerclage technique is shown in Figure 2.

The novel prosthesis body for the TendoClip fixation was specially manufactured for this application (Figure 1). The TendoClip consists of two titanium plates that correspond to the average size of the lesser and greater tuberosity, and the tuberosity is clamped between them like a sandwich. The plates are then fixed to the metaphyseal prosthesis body with the inserted tuberosity using a central titanium screw. The TendoClip plates are made of textured, rough titanium on the bone side for better adhesion. A central hole for the fixation screw must be drilled into the fragment to fix the tuberosity using the sandwich method.

### 2.3 Biomechanical Analysis

**Figure 3:**
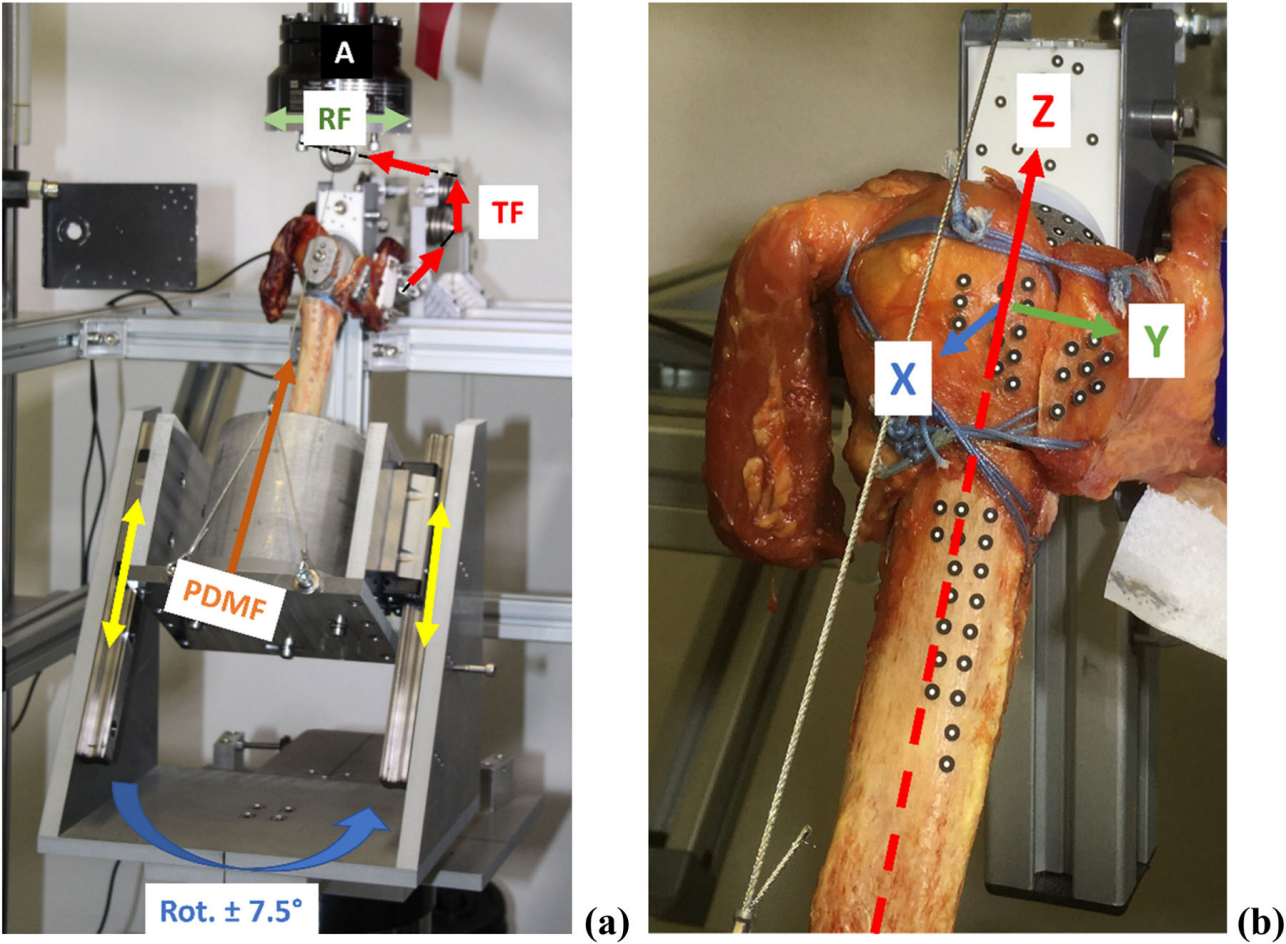
(a) Test setup in servo‐hydraulic testing machine: embedded humerus shaft is brought into contact with the glenosphere through passive deltoid muscle force (PDMF) through sliding bars (/). Tendon force (TF) was generated from rotational forces (RF) of the actuator (A) via tensioned ropes over pulleys. (b) Coordinate system with the center of rotation in the humeral head.

The humeral shaft was initially shortened and cast in a defined mold using synthetic resin (RenCast FC 53, Huntsman, Advanced Materials, Germany). The specimen was therefore fixed 10 mm below the tip of the stem. For the biomechanical investigation of treatment stability, muscle forces of the inserting muscle groups must be simulated. The technical configuration was adapted from previous studies conducted by Schmalzl et al. [21] and Knierzinger et al. [23]. The specimens were placed in a servo‐hydraulic testing machine (MTS 858 Mini Bionix II, MTS Systems Corporation, USA) at 30° abduction on a bearing table, allowing for rotational movements of the base plate of ±7.5° (Figure 3a). The prosthesis body with the inserted inlay was brought into contact with the glenosphere through a passive deltoid muscle force with a dead weight of 1 kg. This aligns the prosthesis′s center of rotation with the testing machine′s axis of rotation. The muscle tendons of the m. subscapularis and m. infraspinatus were fixed with special 3D‐printed clamps. They were connected to the testing machine via steel cables. Tensile forces can be generated in the muscle tendons by redirecting the cables to the rotary drive of the testing machine. Muscle tendons were loaded with a preload of 10 N before the test was started to prevent the cables from slacking.

A cyclical load was chosen to determine the primary stability of the different treatments. A torque of ± 1 Nm was applied to the muscle tendons in the first load stage. This torque results in a pulling force at the tuberosity of 50 N. The torque was increased stepwise by ± 0.25 Nm (corresponding to 10 N) after 100 load cycles. One load cycle lasts 15 s. A total of 10 load stages were run through up to a torque of ± 3.25 Nm (140 N). Failure limits were defined by the maximal angle of rotation of the actuator (± 60°) or failure to reach the target torque of the load level within the specified time.

Relative motion between the tuberosities and the humeral shaft was used to indicate primary stability. The three‐dimensional relative motion was measured using optical, camera‐based measurement techniques (PONTOS‐GOM – Gesellschaft für Optische Messtechnik GmbH, Braunschweig, Germany). Bone and implant makers were applied to the fracture borders of the tuberosities, on the humeral shaft of the specimen and on the prosthesis body (Figure 3b). The maximum relative motion was analyzed in relation to all three spatial axes, with the z‐axis reflected to the humeral shaft axis. The y‐axis was set vertically to the z‐axis, pointing from anterior to posterior. The x‐axis crossed the y‐axis vertically, pointing from medial to lateral (Figure 3b). Relative motion between the components was measured at cycles 10, 50, and 90 for each load level.

### 2.4 Statistical Analysis

Before the start of the experimental study, a sample size calculation was performed using G*Power 3.1.5 (University Kiel, Germany) based on the reported data by Schmalzl et al. [21]. Input parameters to compute the required sample size were tails: two, effect size dz: 1.63, α err prob: 0.05 and power (1‐β err prob): 0.95. This results in the output parameters sample size 8 for each group and an actual power of 0.97.

Statistical analyses were performed using SPSS 25 (IMB, Armonk, NY, USA) with a significance level of *p* < 0.05. Empirical distribution of the measured parameters is reported with mean value, standard deviation, range, and confidence intervals (CIs). Before analysis, the normal distribution of the data was evaluated using a Shapiro–Wilk test, and the homogeneity of variance was verified using the Levene test. *P*‐values are seen as descriptive statistics and have no confirmatory value.

Relative motion was compared with a single‐factor analysis of covariance with repeated measures, and bone density as a continuous covariate was performed. Different measurement times were used as within‐subject factors and the investigated treatment techniques as between‐subject factors. For significant differences regarding between‐subject factors, a post‐hoc test with Bonferroni correction was used to check whether significant differences existed between measurement times with regard to the relative motion. A *t*‐test for dependent samples was used to analyze the maximum force of the two types of treatment.

## 3 Results

The statistical analysis included 7 fresh‐frozen paired human humeri thawed for testing and preparation. One pair was excluded from the relative motion analysis due to a loosening of the prosthesis during the test preparations.

### 3.1 Bone Mineral Density

Density differences between the cerclage group (0.57 g/cm² (SD 0.19)) and TendoClip group (0.56 g/cm² (SD 0.14)) were tested using the Shapiro–Wilk test and showed a normal distribution (*p* = 0.806). The following paired *t*‐test showed no significant differences in bone density between the two groups (*t*(7) = 0.191, *p* = 0.854, *d* = 0.69).

### 3.2 Testing Cycles and Reason of Failure

**Figure 4:**
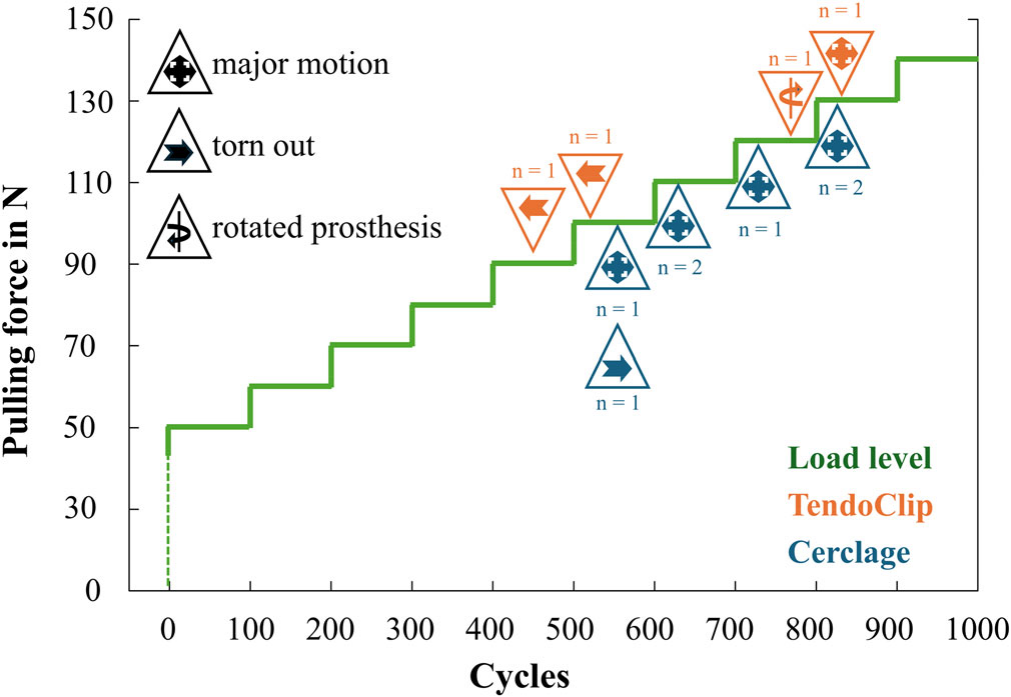
Reason of failure depending on pulling force and reached testing cycles.

The cerclage group reached 675 ± 100 cycles and the TendoClip group reached 790 ± 228 cycles before any fixation failure occurred (*p* = 0.299). Figure 4 shows the reasons for failure depending on the load stages of both groups.

All specimens in the cerclage group resisted a pulling force of 100 N, with 2 even achieving 120 N. In the TendoClip group, all preparations passed a pulling force of 80 N before failures occurred. Here, 5 resisted a pulling force of 120 N and 3 completed the entire test (140 N).

In the cerclage group, the main reason for failure was a major fragment motion of the tuberosities (< 2 mm) (*n* = 6). In one of the preparations, the tendon tore out of the clamps. In the TendoClip group, the tuberosities were torn out of the TendoClips in two treatments. Furthermore, the limit of maximum fragment motion was reached (*n* = 1) and the prosthesis rotated in the humeral shaft (< 2°) (*n* = 1).

### 3.3 Relative Motion

**Figure 5:**
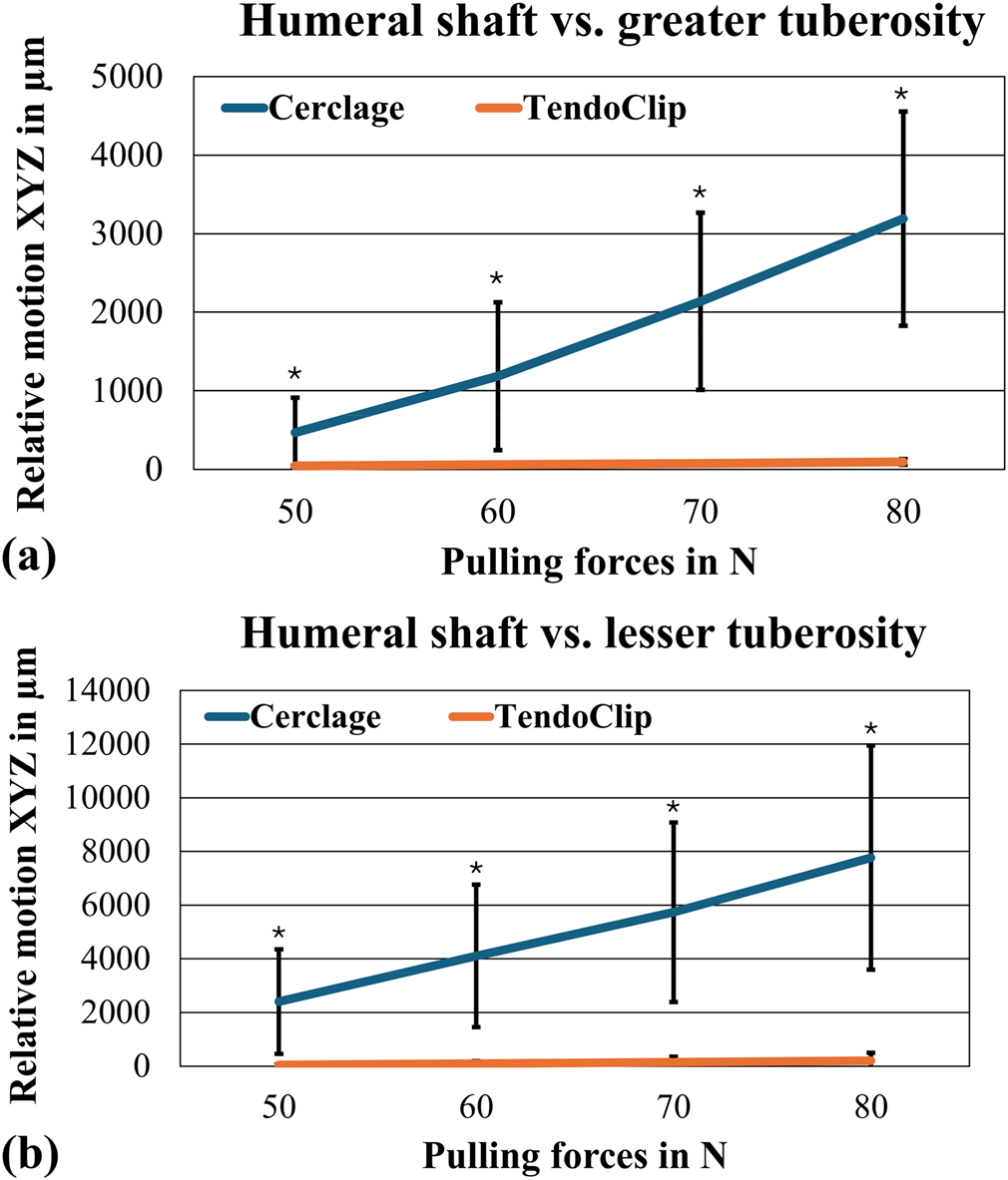
Resulting maximum relative motion (XYZ) as a function of increased load levels for (a) the greater tuberosity and (b) the lesser tuberosity. *Significant differences (*p* < 0.05).

The first four load levels were included to evaluate the maximum relative motions between the humeral shaft and the tuberosities, as all the preparations have passed through the full cycles in this load level. The main direction of fragment motion is in the x‐axis for the greater tuberosities. In the y‐axis, it is for the lesser tuberosities. This corresponds to the main direction of tension through the treatment and the tendon cables on the tuberosities. Figure 4 illustrates the resulting maximum relative motion for the greater (Figure 5a) and lesser (Figure 5b) tuberosity as a function of both groups and incrementally increased pulling forces (50–80 N).

For the motion of the greater tuberosity relative to the humeral stem, increasing pulling forces showed a statistically significant difference in relative motion in both groups (cerclage group: F(1.42, 8.53) = 24.37, *p* < 0.001; TendoClip group: F(5.97, 10.18) = 5.97, *p* = 0.022)) (Table 1). Data in all four load levels was normally distributed (α = 0.05). The dependent‐samples *t*‐test showed significant differences in maximum relative motion at each of the four load levels.

**Table 1:** Resulting (XYZ) maximum relative motion between the tuberosities and the humeral stem for both groups and the first four load levels.

		Greater tuberosity		Lesser tuberosity	
Load level (*N*)	*N* = 7	Mean values ± SD (µm)	*p*‐value	Mean values ± SD (µm)	*p*‐value
1 (50)	Cerclage	465.9 ± 445.8	0.042*	2401.3 ± 1947.7	0.019*
	TendoClip	42.4 ± 15.2		47.0 ± 13.3	
2 (60)	Cerclage	1186.7 ± 940.9	0.020*	4106.6 ± 2652.0	0.008*
	TendoClip	58.9 ± 32.5		92.0 ± 99.7	
3 (70)	Cerclage	2138.6 ± 1127.4	0.003*	5737.2 ± 3344.4	0.005*
	TendoClip	73.3 ± 28.9		147.7 ± 208.3	
4 (80)	Cerclage	3189.5 ± 1363.9	< 0.001*	7769.8 ± 4178.1	0.003*
	TendoClip	93.7 ± 45.6		204.0 ± 292.6	

The analysis of the smaller tuberosity also showed normally distributed data from the second load level onwards (α = 0.05). The dependent variable t‐test shows a statistically significant difference between the two treatment groups in all four load levels. Increasing pulling forces exclusively had a statistically significant influence on the relative motion between the lesser tuberosity and the humeral stem in the cerclage group (F(1.12, 6.72) = 19.38, *p* = 0.003). There was no significant difference in the TendoClip group (F(1.01, 6.07) = 2.03, *p* = 0.203) (Table 1).

**Figure 6:**
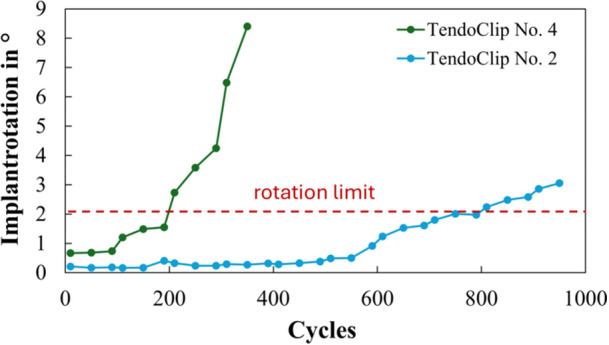
Rotation of non‐fixed implanted stems around the z‐axis.

The motion of the humeral stem in the humeral shaft should be low with optimal press‐fit implantation. Any loosening of the implanted stem is reflected in a rotation around the z‐axis between the humeral shaft and the implanted stem. Figure 6 shows the rotation of two implanted stems as a function of the rotation around the z‐axis over the biomechanical test cycles. In all included specimens from both groups, the rotation of the implanted stem was less than 2°.

## 4 Discussion

Stable fixation of the tuberosities for treating proximal humerus fractures is decisive for the success of bony consolidation and for a positive clinical therapeutic outcome [14, 15, 28]. This study investigates the primary stability of a cerclage fixation technique and the novel TendoClip procedure for fixing the tuberosity fragments after 4‐part PHF on reverse shoulder arthroplasty. One indicator for stability is movement of the tuberosity fragments. The results of the biomechanical investigation show increased relative motion of the fixed tuberosities in the cerclage group in relation to the humeral shaft compared to the TendoClip group. Overall, a higher primary stability of the fixed tuberosity fragments was observed with the TendoClip procedure. This is confirmed by the measured relative motions between the lesser tuberosity and humeral shaft, which were significantly higher in the cerclage group than in the TendoClip group within the first four load levels (*p* = 0.003–0.019). The same could be observed for the greater tuberosity and humeral shaft (*p* = 0.001–0.042). The first cases of failure occurred in both groups when pulling forces above 120 N were applied. For the individual measurements, it should be noted that only 2 specimens in the cerclage group resisted pulling forces of 120 N. In contrast, 3 specimens in the TendoClip group were tested over the entire test without failing. The magnitude of the relative motion measured within the load levels also indicates that the TendoClip procedure ensures a significantly more stable fixation of the tuberosity fragments. The difference in relative motion of the greater tuberosity to the humeral shaft was over ten times higher, even at the first load level (TendoClip 42.4 ± 15.2 µm, cerclage 465.9 ± 445.8 µm). In addition, the nonsignificant increase in relative motion with increasing force level for the lesser tuberosity indicates that a more rigid attachment of the bone fragments to the prosthesis body could be achieved with a TendoClip treatment.

Previous research in the field of tuberosity fixation after 4‐part PHF has focused on comparing the primary stability of different cerclage fixation techniques with cerclage materials. A comprehensive review of the scientific databases revealed that a novel procedure, such as the TendoClip, has not yet been considered alongside the conventional fixation procedure. Dietz et al. [29] investigated the influence of different fixation materials (wire vs. suture) in the tuberosity fixation method on primary stability using a biomechanical model under cyclic tensile forces of up to 40 N. The maximum dislocation of the greater tuberosities in both treatment groups (wire: 0.64 mm; suture: 3.30 mm) was already evident after 4 cycles. Relative motions of the tuberosities in relation to the humeral shaft were significantly greater in the suture cerclage group than in the wire cerclage group. Another cadaver study by Borowsky et al. [30] concluded that suture cerclage has mechanical deficiencies. These are reflected in the suture cutting into bone and tendon material and in the force‐induced elongation of the suture material. Under defined cyclic loads with forces up to 175 N under internal rotation and 87.5 N under external rotation, all tuberosity fragments dislocated and could be moved by at least 10 mm in most cases. The research group of Grubhofer et al. [31] compared the primary stability of two different suture cerclage techniques in a similar test scenario. The treatment of 4‐part PHF with the double‐thread technique, also known as the “cow hitch”, and with the conventional thread technique were subjected to cyclic loading under tensile forces between 250 N and 350 N. Only the relative motions of the greater tuberosity in relation to the humeral shaft were considered in the evaluation. In tests by Grubhofer et al., fracture treatment using the “cow hitch” technique resulted in significantly less dislocation of the greater tuberosity in relation to the humeral shaft (0.73 mm ± 0.33 mm) compared to the conventional cerclage technique (2.28 mm ± 1.08 mm). Research by Knierzinger et. al [23] used a test setup similar to the present study to compare primary stability between suture and wire cerclage. The specimens were subjected to cyclically increasing rotational loads, starting with tensile forces of 20 N. Loads were gradually increased by 5 N until failure occurred. The evaluation criterion used was rotation of the tuberosities in relation to the shaft axis. The first occurrence of the specified limits of 15° rotation occurred at tensile forces of 65 N. On average, the wire cerclage group resisted a tensile load of 90 N. A comparison of the relative motion of the greater tuberosity under suture cerclage with corresponding tensile forces in this study showed that these are lower in comparison to Dietz et al. (3.30 mm vs. 0.91 mm (40 N)) and Borowsky et al. (10 mm vs. 4.55 mm (87.5 N)). In contrast, the measured relative motions of the TendoClip group in our study are significantly lower compared to those of the suture cerclage and wire cerclage groups in Knierzinger et. al. From this, it can be concluded that the primary stability is higher with the TendoClip.

A relevant limitation of the TendoClip procedure is the screwing of the bone fragments to the prosthesis body. The tubercle fragments must be drilled through so that they can be screwed to the prosthesis. The drilling weakens the already small bone fragments and may contribute to difficulties in adequately securing these. During this study, bony avulsion of the lesser tuberosity occurred in two cases. In the author′s opinion, carefully clamping the entire surface without drilling through the fragment could help to improve the treatment. However, clamping of the tuberosities also carries the risk of compression necrosis caused by excessive pressure on the bone fragments. This could lead to destruction of the bone and could be a possible factor in early implant failure. In addition, the predetermined positioning of the tubercle due to the screw connection can lead to significant gaps between the bony parts during restoration. In everyday clinical practice, this would rule out the possibility of bony consolidation. These gaps could be closed with bone substitute materials. However, direct contact after the restoration would still be preferable. Another limitation of the system is the loosening of the prosthesis in the humeral shaft. This was clearly observed in two cases in this study. The rotational force applied on the prostheses caused the prosthesis stem to rotate in the humeral shaft. This phenomenon did not occur with a suture cerclage. This is possibly due to differences in the fixation mechanisms In the cerclage procedure, the tuberosity fragments are held to the fracture body by sutures. The force exerted on the prosthesis by pulling on the fragments is lower than in the TendoClip procedure, in which higher retention forces can be generated on the prosthesis body by screw clamping. Nevertheless, the treatment of a 4‐part PHF with a reattachment of the tuberosities with TendoClips provided promising results for primary stability compared to the cerclage procedure used. The knowledge gained could be used to improve the product before market approval. This should be supplemented by further biomechanical investigations. The TendoClip could represent a standardizable, reproducible procedure for use in everyday clinical practice to support patients starting postoperative rehabilitation at an early stage.

### 4.1 Limitations

Biological bone healing cannot be taken into account in this cadaveric study. Even though our experimental setup reproduced the clinical situation as realistically as possible, the physiological effects of surrounding soft tissues, bone density differences, and present 4‐part PHF could not be reproduced. This limits the extrapolation of our results to the clinical scenario. Another limitation is the representation of a simplified load situation. In this study, we focused on rotational loading of the shoulder joint. Other shoulder joint movement patterns, such as abduction or flexion, were not considered. It can be assumed that a refixed supraspinatus tendon would also affect the primary stability of the fixation technique. Due to the failure of one preparation, the calculated sample size for the statistical analysis of the relative motion could not be achieved. However, as the results are highly evident, this probably does not influence the statements made.

## 5 Conclusion

In this study, the relative motions of individual fracture fragments of a 4‐part proximal humerus fracture to the shaft of the humerus were measured as an indicator of primary stability. Applying tensile forces resulted in a significant difference in the relative motions between the TendoClip and the cerclage group. The experimental investigations showed that 3 out of 8 specimens in the TendoClip group could be loaded for the entire test with pulling forces of 140 N. In the cerclage group, none of the preparations passed the test completely. The recorded failure mechanisms also showed that fixed bone fragments of the cerclage procedure were significantly more frequently affected by loosening and visible relative motion compared to the TendoClip procedure. However, the TendoClip restoration has some limitations as well. In 2 out of 8 cases, a bone fragment was pulled out of the fixation. A possible cause of this is the weakening of the bone fragments when they were prepared for fixation by drilling a hole through them to be fixed to the prosthesis body. In the future, this tear‐out could be minimized by modifying the design of the prosthesis body. The observed stem loosening of the prosthesis in the TendoClip group also needs to be investigated because there might be a connection between loosening and fixation method, as the loosening only occurred in the TendoClip group.

In summary, the data collected show that using the TendoClip reduces bone‐fragment micromotion for 4‐fragment fractures of the proximal humerus. Further investigations of the treatment procedure, such as longer loading times, higher force application, and consideration of further muscle tractions, would be helpful in comprehensively demonstrating the performance of the TendoClip procedure. In conclusion, the TendoClip may represent an alternative to conventional fixation procedures for multifragmentary proximal humerus fractures.

## Author Contributions

Maximilian Uhler: 1, 2, 3. Thilo Patzer: 1,2. Jörg Fleischer: 1. Daniel Knopp: 1. Kevin Knappe: 1. Matthias Bühlhoff: 2, 3. Jan Philippe Kretzer: 1, 2, 3. All listed authors have contributed to the submitted manuscript, agree on the submission and believe that the manuscript represents honest work. The manuscript has not been published previously and is not under consideration for publication elsewhere.

## Disclosure

The authors, their immediate family and any research foundation with which they are affiliated have not received any financial payments or other benefits from any commercial entity related to the subject of this article.

## References

[1] C. Bergdahl , C. Ekholm , D. Wennergren , F. Nilsson , and M. Möller , “Epidemiology and Patho‐Anatomical Pattern of 2,011 Humeral Fractures: Data From the Swedish Fracture Register,” BMC Musculoskeletal Disorders 17 (2016): 159. 27072511 10.1186/s12891-016-1009-8 PMC4830043

[2] S. Iglesias‐Rodríguez , D. M. Domínguez‐Prado , A. García‐Reza , et al., “Epidemiology of Proximal Humerus Fractures,” Journal of Orthopaedic Surgery and Research 16 (2021): 402. 34158100 10.1186/s13018-021-02551-x PMC8220679

[3] C. S. Neer, 2nd , “Displaced Proximal Humeral Fractures,” Journal of Bone & Joint Surgery 52 (1970): 1077–1089. 5455339

[4] S. Rosas , T. Y. Law , J. Kurowicki , N. Formaini , S. P. Kalandiak , and J. C. Levy , “Trends in Surgical Management of Proximal Humeral Fractures in the Medicare Population: A Nationwide Study of Records From 2009 to 2012,” Journal of Shoulder and Elbow Surgery 25 (2016): 608–613. 26475637 10.1016/j.jse.2015.08.011

[5] A. Mata‐Fink , M. Meinke , C. Jones , B. Kim , and J. E. Bell , “Reverse Shoulder Arthroplasty for Treatment of Proximal Humeral Fractures in Older Adults: A Systematic Review,” Journal of Shoulder and Elbow Surgery 22 (2013): 1737–1748. 24246529 10.1016/j.jse.2013.08.021

[6] J. R. Ferrel , T. Q. Trinh , and R. A. Fischer , “Reverse Total Shoulder Arthroplasty Versus Hemiarthroplasty for Proximal Humeral Fractures: A Systematic Review,” Journal of Orthopaedic Trauma 29 (2015): 60–68. 25186842 10.1097/BOT.0000000000000224

[7] D. Gallinet , X. Ohl , L. Decroocq , C. Dib , P. Valenti , and P. Boileau , “Is Reverse Total Shoulder Arthroplasty More Effective Than Hemiarthroplasty for Treating Displaced Proximal Humerus Fractures in Older Adults? A Systematic Review and Meta‐Analysis,” Orthopaedics & Traumatology: Surgery & Research 104 (2018): 759–766. 10.1016/j.otsr.2018.04.025 29969722

[8] D. Gallinet , A. Adam , N. Gasse , S. Rochet , and L. Obert , “Improvement in Shoulder Rotation in Complex Shoulder Fractures Treated by Reverse Shoulder Arthroplasty,” Journal of Shoulder and Elbow Surgery 22 (2013): 38–44. 22705317 10.1016/j.jse.2012.03.011

[9] D. Gallinet , P. Clappaz , P. Garbuio , Y. Tropet , and L. Obert , “Three or Four Parts Complex Proximal Humerus Fractures: Hemiarthroplasty Versus Reverse Prosthesis: A Comparative Study of 40 Cases,” Orthopaedics & Traumatology: Surgery & Research 95 (2009): 48–55. 10.1016/j.otsr.2008.09.002 19251237

[10] E. Sebastiá‐Forcada , R. Cebrián‐Gómez , A. Lizaur‐Utrilla , and V. Gil‐Guillén , “Reverse Shoulder Arthroplasty Versus Hemiarthroplasty for Acute Proximal Humeral Fractures. A Blinded, Randomized, Controlled, Prospective Study,” Journal of Shoulder and Elbow Surgery 23 (2014): 1419–1426. 25086490 10.1016/j.jse.2014.06.035

[11] D. J. Cuff and D. R. Pupello , “Comparison of Hemiarthroplasty and Reverse Shoulder Arthroplasty for the Treatment of Proximal Humeral Fractures in Elderly Patients,” Journal of Bone and Joint Surgery 95 (2013): 2050–2055. 10.2106/JBJS.L.01637 24257664

[12] J. L. Berliner , A. Regalado‐Magdos , C. B. Ma , and B. T. Feeley , “Biomechanics of Reverse Total Shoulder Arthroplasty,” Journal of Shoulder and Elbow Surgery 24 (2015): 150–160. 25441574 10.1016/j.jse.2014.08.003

[13] M. F. Pastor , M. Kraemer , M. Wellmann , C. Hurschler , and T. Smith , “Anterior Stability of the Reverse Shoulder Arthroplasty Depending on Implant Configuration and Rotator Cuff Condition,” Archives of Orthopaedic and Trauma Surgery 136 (2016): 1513–1519. 27566617 10.1007/s00402-016-2560-3

[14] D. Gallinet , J. F. Cazeneuve , E. Boyer , et al., “Reverse Shoulder Arthroplasty for Recent Proximal Humerus Fractures: Outcomes in 422 Cases,” Orthopaedics & Traumatology: Surgery & Research 105 (2019): 805–811. 10.1016/j.otsr.2019.03.019 31279769

[15] N. P. Jain , S. S. Mannan , R. Dharmarajan , and A. Rangan , “Tuberosity Healing After Reverse Shoulder Arthroplasty for Complex Proximal Humeral Fractures in Elderly Patients‐Does It Improve Outcomes? A Systematic Review and Meta‐Analysis,” Journal of Shoulder and Elbow Surgery 28 (2019): e78–e91. 30593437 10.1016/j.jse.2018.09.006

[16] J. Schmalzl , M. Jessen , M. Holschen , et al., “Tuberosity Healing Improves Functional Outcome Following Primary Reverse Shoulder Arthroplasty for Proximal Humeral Fractures with a 135° Prosthesis,” European Journal of Orthopaedic Surgery & Traumatology 30 (2020): 909–916. 32162048 10.1007/s00590-020-02649-8

[17] T. Bufquin , A. Hersan , L. Hubert , and P. Massin , “Reverse Shoulder Arthroplasty for the Treatment of Three‐ and Four‐Part Fractures of the Proximal Humerus in the Elderly: A Prospective Review of 43 Cases with a Short‐Term Follow‐Up,” Journal of Bone and Joint Surgery. British Volume 89 (2007): 516–520. 17463122 10.1302/0301-620X.89B4.18435

[18] C. M. Jobin , B. Galdi , O. A. Anakwenze , C. S. Ahmad , and W. N. Levine , “Reverse Shoulder Arthroplasty for the Management of Proximal Humerus Fractures,” Journal of the American Academy of Orthopaedic Surgeons 23 (2015): 190–201. 25630370 10.5435/JAAOS-D-13-00190

[19] J. Ménard, Jr. , M. Émard , F. Canet , V. Brailovski , Y. Petit , and G. Y. Laflamme , “Initial Tension Loss in Cerclage Cables,” Journal of Arthroplasty 28 (2013): 1509–1512. 23618753 10.1016/j.arth.2013.03.014

[20] P. J. Denard , P. C. Nolte , P. J. Millett , et al., “A Tensionable Suture‐Based Cerclage Is an Alternative to Stainless Steel Cerclage Fixation for Stabilization of a Humeral Osteotomy During Shoulder Arthroplasty,” Journal of the American Academy of Orthopaedic Surgeons 29 (2021): e609–e617. 32947346 10.5435/JAAOS-D-20-00047

[21] J. Schmalzl , M. Piepenbrink , J. Buchner , S. Picht , C. Gerhardt , and L. J. Lehmann , “Higher Primary Stability of Tuberosity Fixation in Reverse Fracture Arthroplasty with 135° Than with 155° Humeral Inclination,” Journal of Shoulder and Elbow Surgery 30 (2021): 1257–1265. 33010438 10.1016/j.jse.2020.09.009

[22] J. Schmalzl , M. Piepenbrink , J. Buchner , S. Picht , C. Gerhardt , and L. J. Lehmann , “Tensioning Device Increases Biomechanical Stability of Tuberosity Fixation Technique With Cerclage Sutures in Reverse Shoulder Arthroplasty for Fracture,” Journal of Shoulder and Elbow Surgery 30 (2021): 1214–1221. 32871265 10.1016/j.jse.2020.08.015

[23] D. Knierzinger , C. H. Heinrichs , C. Hengg , M. Konschake , F. Kralinger , and W. Schmoelz , “Biomechanical Evaluation of Cable and Suture Cerclages for Tuberosity Reattachment in a 4‐Part Proximal Humeral Fracture Model Treated with Reverse Shoulder Arthroplasty,” Journal of Shoulder and Elbow Surgery 27 (2018): 1816–1823. 29779978 10.1016/j.jse.2018.04.003

[24] J. Fleischer , A. Schleyer , R. Nassutt , U. Grittner , I. Ojodu , and S. J. Hopp , “Biomechanical Strength and Failure Mechanism of Different Tubercula Refixation Methods Within the Framework of an Arthroplasty for Shoulder Fracture,” Orthopaedics & Traumatology: Surgery & Research 103 (2017): 165–169. 10.1016/j.otsr.2016.12.001 28093375

[25] F. Linde and H. C. F. Sørensen , “The Effect of Different Storage Methods on the Mechanical Properties of Trabecular Bone,” Journal of Biomechanics 26 (1993): 1249–1252. 8253829 10.1016/0021-9290(93)90072-m

[26] A. M. Doetsch , J. Faber , N. Lynnerup , et al., “Bone Mineral Density Measurement Over the Shoulder Region,” Calcified Tissue International 71 (2002): 308–314. 12170375 10.1007/s00223-001-2082-y

[27] P. Boileau , G. Alami , A. Rumian , D. G. Schwartz , C. Trojani , and A. J. Seidl , “The Doubled‐Suture Nice Knot,” Orthopedics 40 (2017): e382–e386. 27942736 10.3928/01477447-20161202-05

[28] R. W. Simovitch , C. P. Roche , R. B. Jones , et al., “Effect of Tuberosity Healing on Clinical Outcomes in Elderly Patients Treated With a Reverse Shoulder Arthroplasty for 3‐ and 4‐Part Proximal Humerus Fractures,” Journal of Orthopaedic Trauma 33 (2019): e39–e45. 30688837 10.1097/BOT.0000000000001348

[29] S. O. Dietz , S. Kuhn , E. Gercek , et al., “Die Problematik Der Tuberkularefixation in Der Frakturprothetik Des Humeruskopfes,” Obere Extremität 5 (2010): 106–114.

[30] K. A. Borowsky , V. R. Prasad , L. J. Wear , et al., “Is Failure of Tuberosity Suture Repair in Hemi‐Arthroplasty for Fracture Mechanical?,” Journal of Shoulder and Elbow Surgery 22 (2013): 971–978. 23333733 10.1016/j.jse.2012.09.002

[31] F. Grubhofer , E. Bachmann , C. Gerber , et al., “Cow‐Hitch‐Suture Cerclage for Fixation of the Greater Tuberosity in Fracture Rtsa,” JSES International 5 (2021): 270–276. 33681848 10.1016/j.jseint.2020.10.016 PMC7910725

